# Vulcanite Litigation

**Published:** 1874-10

**Authors:** 


### VULCANITE LITIGATION.

We are pleased to print the following statement from the advance sheet of the *Dental Cosmos.* It is a statement so clear that no one can misunderstand it. We commend it to the careful attention of every one interested:

"Since our last mention of the of the suit against Dr. Smith, and its decision by Judge Shepley, the final decree has been entered in conformity with the proceedings taken thereupon, and this decree bears date August 18th, 1874.

Since that time all the necessary formalities have been complied with (the supersedeas bond filed, etc.,) and an appeal to the Supreme Court of the United States duly perfected, thus arresting the execution of the judgment against Dr. Smith.

Between the taking of an appeal and the hearing of it, it is well known a considerable time must elapse, because the docket of the Supreme Court is always a long one, and cases as a rule are heard in numerical order. We have therefore only to wait until Dr. Smith's turn comes. When a case is argued the Court decides it very promptly. Nothing can be done to to hurry it, and it only needs that the profession bear in mind the foregoing facts whenever any suggestion occurs as to why the appeal is not heard of for a season.

At this point we deem it proper to add a word. During the somewhat similar interval that has elapsed since the decision by Judge Shepley, all sorts of assertions, statements, and surmises have been made or indulged, and have reached us through divers channels, coming from parties interested in one way or another, and having an object or purpose that could readily be comprehended. The word we wish to add is simply that since the decision by Judge Shepley nothing has been done in the Smith case, except with a view to obtaining the final decree on the one side, and the securing of an appeal on the other; and in the latter proceeding the ten days allowed by law, from August 18th, were more than sufficient. Thus at the earliest practical moment the case has been placed in train for ultimate disposition, and the programme originally contemplated, in a stipulation adopting this as a test case, fully and promptly carried out up to date.

Furthermore, in regard to the cases in other circuits where Judge Shepley's opinion has been adopted and acted upon, this also was contemplated in the acceptance of a test case and the proceedings elsewhere, in what may be called these collateral cases, have simply been the natural and expected consequences of the Massachusetts decision.

As we understand it, the Michigan cases are governed by a specific stipulation applying exclusively to them (as the original stipulation in the Smith case applied only to the Pennsylvania, New Jersey, and Delaware cases) and are awaiting a hearing in Michigan (as the other cases awaited a hearing before Judge Shepley,) all further proccedings in that circuit being suspended until such hearing and decision.

Since the appeal of the Gardner suit was dismissed and the mandate recalled by the United States Stpreme Court, on the ground that that suit both below and above was *merely collusive*, the conduct of the defense has been in harmony with the purpose resulting from the desire then expressed by leading men in the dental profession that the undersigned would take charge of the preparation, illustration and presentation of the processes, and products of those processes, which had been in public use and on sale by the dental profession long before the alleged claims of invention by Dr. Cummings, and which had so strangely been omitted from the Gardner record, although their effect has been at least to change the character of the patent entirely as now construed both by the Company and the Court. The Goodyear Dental Vulcanite Company declining to argue the original cases in this circuit, permitting their preliminary suits to go by, paying the costs and renewing them again, their offer to make the Smith case the test case was accepted, as affording the earliest opportunity of having the matter heard on its merits.

The case having been fully presented before a Circuit Court and having now been appealed to the Supreme Court without delay, will be finally argued there in its turn.

It will therefore be evident to the profession that there has been no disposition to provoke litigation, and no litigation resorted to further than was necessary to present the facts properly for conclusive adjudication." SAMUEL S. WHITE.

